# Cost-Effectiveness of Improved Hypertension Management in India through Increased Treatment Coverage and Adherence: A Mathematical Modeling Study

**DOI:** 10.5334/gh.952

**Published:** 2021-05-10

**Authors:** Hemanshu Das, Andrew E. Moran, Anupam K. Pathni, Bhawna Sharma, Abhishek Kunwar, Sarang Deo

**Affiliations:** 1 Indian School of Business, Hyderabad, IN; 2 Max Institute of Healhcare Management, Sahibzada Ajit Singh Nagar, IN; 3 Resolve to Save Lives, an initiative of Vital Strategies, New York, US; 4 Division of General Medicine, Columbia University Irving Medical Center, New York, US; 5 World Health Organization Country Office for India, New Delhi, IN

**Keywords:** hypertension, cost-effectiveness, developing countries, health policy, preventive care

## Abstract

**Background:**

Despite the availability of effective and affordable treatments, only 14% of hypertensive Indians have controlled blood pressure. Increased hypertension treatment coverage (the proportion of individuals initiated on treatment) and adherence (proportion of patients taking medicines as recommended) promise population health gains. However, governments and other payers will not invest in a large-scale hypertension control program unless it is both affordable and effective.

**Objective:**

To investigate if a national hypertension control intervention implemented across the private and public sector facilities in India could save overall costs of CVD prevention and treatment.

**Methods:**

We developed a discrete-time microsimulation model to assess the cost-effectiveness of population-level hypertension control intervention in India for combinations of treatment coverage and adherence targets. Input clinical parameters specific to India were obtained from large-scale surveys such as the Global Burden of Disease as well as local clinical trials. Input hypertensive medication cost parameters were based on government contracts. The model projected antihypertensive treatment costs, avoided CVD care costs, changes in disability-adjusted life year (DALYs) and incremental cost per DALY averted (represented as incremental cost-effectiveness ratio or ICER) over 20 years.

**Results:**

Over 20 years, at 70% coverage and adherence, the hypertension control intervention would avert 1.68% DALYs and be cost-saving overall. Increasing adherence (while keeping coverage constant) resulted in greater improvement in cost savings compared to increasing coverage (while keeping adherence constant). Results were most sensitive to the cost of antihypertensive medication, but the intervention remained highly cost-effective under all one-way sensitivity analyses.

**Conclusion:**

A national hypertension control intervention in India would most likely be budget neutral or cost-saving if the intervention can achieve and maintain high levels of both treatment coverage and adherence.

## Introduction

Globally, cardiovascular diseases (CVDs) account for 31% of all deaths, 80% of which occur in low- and middle-income countries (LMICs) [1]. India, one of the largest LMICs, is estimated to have suffered an economic loss of $94 billion due to CVDs in 2017 [2]. In the last 20 years, the annual incidence of CVDs has almost doubled to 7.4 million, and 60 million Indians are currently living with CVDs [3]. The burden of CVDs in India is projected to increase further due to population growth and aging, which could jeopardize the recent progress in poverty reduction [2,4].

High blood pressure is the single largest preventable risk factor for CVDs and is estimated to account for more than half of the CVD deaths [5]. Low cost and effective blood-pressure lowering treatments are available, which can reduce the morbidity and mortality associated with hypertension [6]. However, in India, 14% of hypertensive individuals above 50 years of age and 8% of hypertensive individuals younger than 50 have their blood pressure under control, largely due to low awareness of hypertensive status and lack of medication adherence [6,7,8,9,10].

Blood pressure control can be improved by implementing a package of increased hypertension screening and a simplified hypertension treatment protocol, enabled by trained health care workers and a robust health information system [9,11,12,13]. Such an intervention, if scaled to cover 70% of all hypertensive individuals worldwide, could avert 7.4 million CVD-related deaths in the next 25 years [6,14]. Lifestyle modifications such as increased physical activity, reduced sodium and trans-fat intake, and tobacco cessation can further improve health outcomes, but lifestyle modifications were found to be difficult to maintain [15,16]. Thus, several pilot projects for improving antihypertensive care delivery are currently being implemented in India based on the principles of increased status awareness and medication adherence [9,17,18]. However, their focus on public health facilities severely limit their potential impact as more than 80% of the hypertensive patients seek treatment in the private sector, where chronic care is often suboptimal and leads to poor outcomes [10,19,20,21]. Thus, a public health intervention to improve hypertension control must cover both public and private sectors, perhaps through a government-run health insurance program. But, given the government’s financial constraints, this is more likely if the intervention is cost-saving and affordable for the health system and not merely ‘cost-effective’ [22,23].

In our study, we set out to investigate if a national hypertension control intervention implemented across the private and public sector facilities could save overall costs of CVD prevention and treatment. We determine the level of coverage and adherence required for the intervention to be cost-saving. We further investigate the total budgetary resources required for such an intervention and the expected savings in healthcare expenditure due to the prevention of CVDs.

## Methods

### Microsimulation Model

We developed a discrete-time microsimulation model for two hypothetical cohorts comprising of 10,000 individuals each (males and females) of 40–69 years of age at the beginning of the simulation. We calculated the costs and disability-adjusted life years (DALYs) for these cohorts over 20 years using cycle time of one month. The outcome measured was the incremental cost per DALY averted (represented as incremental cost-effectiveness ratio or ICER). Our analysis was conducted from the perspective of the government as a payor that allocates budget for such an intervention.

**Figure 1:**
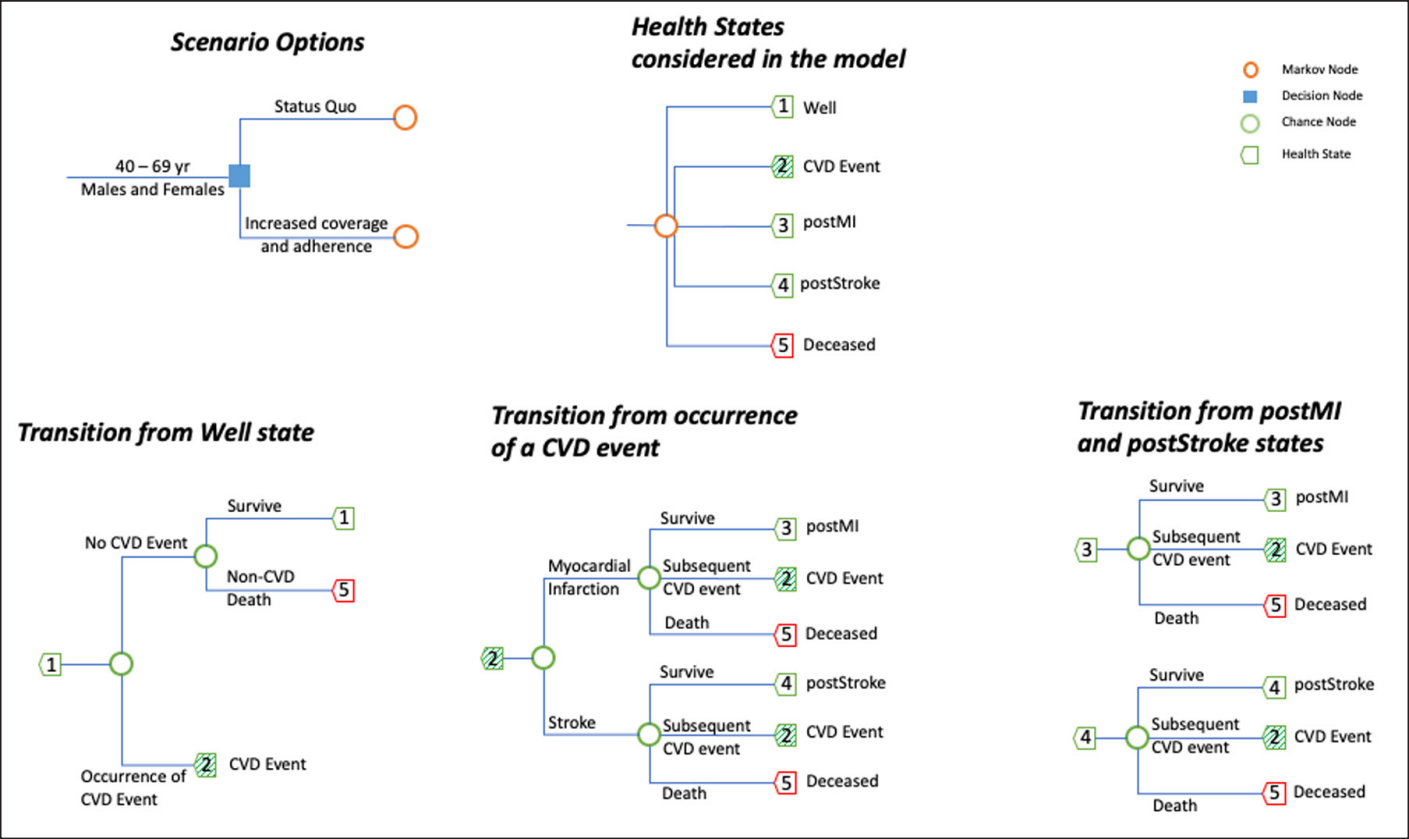
Structure of the microsimulation model. The above microsimulation model was used with a cycle time of one month to estimate costs and DALYs in two hypothetical cohort of 10,000 individuals (males and females respectively) with ages 40-69yr at the start of the simulation. Multiple scenarios with combinations of coverage and adherence were simulated, and the incremental cost per DALY averted was calculated in reference to the status quo of 17% coverage and 30% adherence. The blue square indicates the choice between various intervention scenarios, and the orange circle indicates the chosen intervention. The health states (indicated by green pentagon) comprises of (1) well (no past CVD event), (2) occurrence of a CVD event, (3) surviving post a myocardial infarction (postMI), (4) surviving post a stroke (postStroke), and (5) deceased state. The blue-colored branches from each heath state lead to another heath state based on the probability of the intermediate event (indicated by the green circle). The (2) CVD event is a transitionary markov state and comprises of either an occurrence of MI or stroke. CVD = Cardiovascular disease, MI = Myocardial infarction, DALYs = Disability adjusted life year.

The structure of the model is illustrated in Figure 1. Each individual is at risk for myocardial infarction (MI) or stroke based on their risk factors, which include age, sex, systolic blood pressure, smoking habit, and body mass index. After a first event, individuals with history of MI or stroke (together defined as cardiovascular disease or CVD in our study) face a higher risk of death as well as recurrence of MI or stroke compared to an individual without the history. Individuals on antihypertensive medication experience reduced risk of CVD. We used Python 3.7 to construct the model and perform the analysis.

#### Probability of Clinical Events

We used the WHO Study on global AGEing and adult health (SAGE) dataset to obtain levels of risk factors for the Indian population of relevant ages and used the Globorisk calculator to determine the risk of an MI or stroke based on these risk factors [24,25]. Survival after a CVD event was obtained from the Global Burden of Disease (GBD) 2017 study [26], and all-cause mortality rates were determined using India-specific WHO lifetables [27]. CVD risk reduction due to hypertension treatment was based on age, initial systolic blood pressure (SBP) and medication dosage for each individual [28]. The 30-day risk of death after MI and stroke were derived from local studies [29,30], and the death rate after non-fatal CVD event was calibrated based on GBD data. Probabilities for reinfarction and recurrence of stroke were obtained from published literature [31,32,33]. Selected input values and their sources are shown in Table 1, with the complete list of values provided in Table S2–S7 of the Appendix.

**Table 1:** Select input parameters for the microsimulation model.

Input Parameter	Value	Source(s)

Population characteristics		

Individual profiles consisting of Age, Sex, Systolic blood pressure, BMI and Smoking habit		WHO SAGE [24]
Baseline status-awareness ratio	40.8%	PURE Study [34]
Baseline treatment initiation ratio	77.7%	PURE Study [34]
Baseline treatment persistence ratio	61%	Van Wijk et al. 2005 [35]
Baseline medication compliance ratio	50.3%	Dennis et al. 2011 [36]
Blood pressure increase with increase in age of an individual	Age and initial SBP-specific	Simulated based on model developed by Bellows et al. 2019 [37]
**Mortality and risk of cardiovascular diseases**		

Non-CVD death rate	0.005–0.176 (Age- and sex- specific)^#^	Calculated from WHO lifetables and GBD 2017 [3, 27]
Probability of first-time cardiovascular disease (CVD) event	Individual risk characteristic specific	Obtained from the Globorisk Office Calculator standardized for India [25]
**Acute CVD Events**		

*Myocardial Infarction (MI)*		
Probability of MI if CVD event occurs	37.6– 66.7% (Age- and sex- specific)^#^	Calculated based on GBD 2017 [3]
30-day fatality	0.01–0.13 (Age- and sex– specific)^#^	Calibrated based on findings of Huffman et al. 2018 [29]
Reinfarction (in 30 days)	0.0120 (0.0099–0.0141)ψ	ACS QUIK Study by Huffman et al. 2018 [29]
Acute Stroke (in 30 days)	0.0060 (0.0045–0.0075)ψ	ACS QUIK Study by Huffman et al. 2018 [29]
*Stroke*		
Probability of Stroke if CVD event occurs	33.2–62.3% (Age- and sex- specific)^#^	Calculated based on GBD 2017 [3]
30-day fatality	0.12, 0.13 (Sex-specific)^#^	Calibrated based on a multi-site study by Pandian and Sudhan 2013 [30]
Repeat Stroke (in 30 days)	0.15 (0.1–0.2)ψ	Petty et al. 1998 [32]
**Chronic CVD Events**		

*Ischemic Heart Disease (IHD)*		
Monthly risk of mortality	0.001–0.019 (Age- and sex- specific)^#^	Calibrated based on GBD 2017 [3]
Reinfarction	0.079 (0.073–0.085)ψ	Based on Steg et al. 2007 [31] and derived by Lin et al. 2019 [33]
Acute Stroke	0.014 (0.012–0.016)ψ	Based on Steg et al. 2007 [31], and derived by Lin et al. 2019 [33]
*Stroke*		
Monthly risk of mortality	0.001–0.013 (Age- and sex- specific)^#^	Calibrated based on GBD 2017 [3]
Acute MI	0.043 (0.038–0.048)ψ	Based on Steg et al. 2007 [31], and derived by Lin et al. 2019 [33]
Acute Stroke	0.037 (0.033–0.041)ψ	Based on Steg et al. 2007 [31], and derived by Lin et al. 2019 [33]
Relative risk of fatality for an individual with two or more CVD events	1.5	Smolina et al. 2012 [38]
**Effect of antihypertensive medication**		

Medication protocol for an individual	Initial SBP-specific^#^	Based on India Hypertension Control Initiative (IHCI) implemented in Punjab [39,40]
IHD relative risk due to medication	0.32–0.89 (Age- and initial SBP-specific)^#^	Based on findings by Law et al. 2009 [28]
Stroke relative risk due to medication	0.20–0.89 (Age- and initial SBP-specific)^#^	Based on findings by Law et al. 2009 [28]
IHD relative risk if partially adherent	0.66–0.95 (Age- and initial SBP-specific)	Calculated based on a linear relationship between adherence and efficacy as considered by Cherry et al. 2009 [41]
Stroke relative risk if partially adherent	0.60–0.95 (Age- and initial SBP-specific)	Calculated based on a linear relationship between adherence and efficacy as considered by Cherry et al. 2009 [41]
**Costs**		

Programmatic Cost of Intervention	$0.13 per individual per annum^#^	Calculated from resource costs of India Hypertension Control Initiative
*Antihypertensive treatment*		
Antihypertensive medication (per individual per annum) in public sector	$0.88–17.90 (Drug and dosage specific)^§^	Drug costs based on government rate contracts [42], and the type and dosage drug dispensed is based on the treatment protocol
Antihypertensive medication (per individual per annum) in private sector	$5.42–$125.14 (Drug and dosage specific)^§^	Average cost from Indian online drug retailer 1mg.com [43], and the type and dosage drug dispensed is based on the treatment protocol
Out-patient consultations (per visit)	$1.94 ($1.36–$2.47)^§^	Based on Indian public healthcare sector study by Prinja et al. 2020 [44]
One-time diagnostic tests	$2.27	Government rate contracts [45]
*Acute CVD care*		
In-patient costs for MI	$1040	WHO Choice [46] inflated to 2019–20
In-patient costs for Stroke	$940	WHO Choice [46] inflated to 2019–20
*Chronic CVD care*		
Secondary care medication in public sector (per individual per annum)	$92, $184 (Dosage-specific)^§^	International Drug Price Indicator inflated to 2019–20 [47]
Secondary care medication in private sector (per individual per annum)	$227, $454 (Dosage-specific)^§^	Mean cost from Indian online drug retailer 1mg.com [43]
Outpatient cost for IHD (per annum)	$45	WHO Choice [46] inflated to 2019–20
Outpatient cost for Stroke (per annum)	$67	WHO Choice [46] inflated to 2019–20
**Disability Weights**		

Disutility due to daily medication	0.049 (0.031–0.072)ψ	GBD disability weights [48]
*Acute Events*		
Myocardial Infarction	0.432 (0.288–0.579)ψ	GBD disability weights [48]
Stroke	0.570 (0.377–0.707)ψ	GBD disability weights [48]
Occurrence of second or later CVD event	0.985 (0.992–0.989)ψ	GBD disability weights and Lin et al. 2019 [33]
*Chronic States*		
Ischemic Heart Disease	0.08 (0.02–0.24)ψ	GBD disability weights [48]
Stroke	0.135 (0.01–0.437)ψ	GBD disability weights [48]
Alive post 2+ CVD Events	0.242 (0.11–0.437)ψ	GBD disability weights [48]

The ranges marked with # are further expanded in the Appendix Table S2–5 since the specific value for an individual is based on an individual’s age, sex, and/or SBP. The cost ranges marked with § are based on the specific drug and dosage administered to an individual and has been further expanded in the Appendix Table S6–9. The ranges with the superscript of ψ are 95% confidence intervals with the values sampled based on a β distribution in the simulation runs.

#### Costs

We calculated direct medical costs over 20 years for each individual. These costs include the cost of antihypertensive treatment (outpatient consultation, diagnostic tests, and drugs), acute CVD events (hospital care costs including consultations, room cost, procedures, surgeries, and drugs), and chronic CVD care (outpatient consultations and drugs). We obtained unit costs for antihypertensive medications, chronic CVD care and acute CVD care based on government rate contracts, the International Drug Price Indicator, and WHO CHOICE, respectively [43,46,47]. Programmatic costs are based on the India Hypertension Control Initiative (IHCI), a population-level hypertension control program that has so far enrolled more than 800,000 patients across 25 districts in six states of India [11,49]. This national hypertension-control program aims to increase detection at health facilities by strengthening opportunistic screening, simplifying and standardizing treatment, improving availability of blood pressure monitoring equipment and antihypertensive drugs, and better tracking of patients through a robust information technology platform. The human resources and technology costs to deliver the IHCI program components were determined based on expert interviews and have been factored in as the programmatic cost of the intervention. We used the exchange rate of ₹ 70 to $1 for conversion of costs from Indian rupees to US dollars.

#### Utility Values of Health States

We used disability weights from the GBD study to quantify health loss experienced by individuals who develop fatal or non-fatal CVD [48]. In line with other cost-effectiveness studies, we accounted for loss in wellbeing only. Future costs and DALYs were discounted at 3% per annum.

### Analysis of Coverage and Adherence Scenarios

In the main analysis, we simulated multiple scenarios of hypertension management with varying levels of coverage and adherence. Coverage was defined as the proportion of hypertensive individuals initiated on treatment and was simulated by modifying (i) the proportion of hypertensive individuals who were aware of their blood pressure, and (ii) the proportion of status-aware individuals who were eligible for and initiated on treatment. According to the simplified treatment protocol of IHCI, any individual with SBP ≥ 140 mmHg on readings from two separate occasions is eligible for treatment [39,40]. We defined adherence as the proportion of individuals initiated on treatment who take medications as prescribed, and was assessed by modifying (i) the proportion of individuals who persist with treatment for more than one year after initiation, and (ii) the proportion of individuals highly compliant to medication interval and dosage (proportion of drugs consumed >80%) among those who persist with treatment for more than one year [50]. We calculated the direct medical cost per patient, and applied it based on the number of patients adherent on treatment every cycle duration. The programmatic cost was calculated at the population level and applied as a constant across different scenarios of coverage and adherence.

We simulated an aspirational scenario of 70% coverage and adherence (referenced as 70% scenario henceforth). The scenario was compared against the prevailing coverage and adherence in India, which we termed as *status quo*. The status quo was simulated with a coverage of 17%, an adherence of 30%, and the National Program for Prevention and Control of Cancer, Diabetes, CVD and Stroke (NPCDCS) treatment guideline (individuals are initiated on treatment if either SBP ≥ 180 mmHg or 10-yr CVD risk > 20%) [34,35,36,51,52]. We simulated the 70% scenario because it has been proposed as an immediately achievable target that national programs should aspire [6]. Additionally, we analyzed scenarios with coverage of 40%, 60%, 80%, and 100% and adherence of 40%, 60%, 80%, and 100%.

### Sensitivity Analysis

We tested the robustness of our results through one-way sensitivity analysis by changing values of key input parameters in a set of independent simulation runs. These included increased medication cost and programmatic cost, reduced baseline CVD risk due to possible overestimation using Globorisk calculator, NPCDCS treatment guideline [52], inclusion of disutility of chronic medication and longer and shorter time horizons. Further we simulated a public private sector mix with 80% of the patients accessing hypertension care in the private sector [19], where the private sector medication cost was based on average listed price from an Indian online drug retailer [43]. We ran our model 1000 times with values of input parameters drawn jointly from their respective distributions and calculated costs and DALYs.

### Threshold for Cost-Effectiveness

All ICERs were reported in 2019 US dollars per DALY averted. Scenarios with an ICER below India’s per-capita GDP (US $2338) were considered as cost-effective and scenarios with ICER below half of India’s per-capita GDP (US $1169) were considered as highly cost-effective [53,54]. The scenarios where the overall spending reduced while DALYs averted increased were considered as cost-saving scenarios, and the ICERs were not calculated for such scenarios.

We recognized the criticism around cost-effectiveness vis-à-vis affordability of a program [22]. Thus, along with ICERs, we also calculated the budget impact and required investment in primary care to substantiate the required resources for a hypertension management initiative. Also, our cost-effectiveness threshold of *per-capita GDP* was stricter and more realistic for India (an LMIC) than WHO’s *three times the per-capita GDP* [55,56].

### Ethics Approval

We used publicly available, de-identified data for our analysis in the study, and thus did not seek approval from institutional review boards. Our study conforms to Consolidated Health Economic Evaluation Reporting Standards guidelines, with the filled checklist available in Table S17 of the Appendix [57].

## Results

### Cost-Effectiveness Analysis of Scenarios

Over 20 years, in the 70% scenario (coverage and adherence at 70%), the intervention was cost-saving among both females and males. The scenario averted 5.85% and 3.78% events and 6.1% and 3.76% deaths among females and males compared to status quo. In this scenario, the increase in per-capita antihypertensive medication cost ($18.5 for females and $13.7 for males) was offset by the decrease in CVD treatment cost (–$22.1 for females and –$16.8 for males). In a threshold analysis, we found that the scenario remained highly cost-effective for an increase in incremental cost of up to 11.8% for every individual in the cohort. We have summarized the result in Table 2, with the detailed results in tables S11–13 of the Appendix.

**Table 2:** Costs and health outcomes associated with 70% coverage and adherence in a hypertension control intervention for individuals between 40–69 yrs from 2020–40

ICER ($/DALY averted, 95% UI)		DALYs Averted (percent, 95% UI)		CVD Events Averted (percent, 95% UI)		Per-capita incremental costs over 20 years^^^		Annual net expenditure^^,ψ^ (in ‘000 US $)	Proba-bility of Cost Saving^#^

Female	Male	Female	Male	Female	Male	Antihypertensive Treatment ($, 95% UI)	CVD Treatment ($, 95% UI)		

Cost-saving	Cost-Saving	2.17 (2.15 to 2.19)	1.30 (1.29 to 1.32)	5.85 (5.82 to 5.89)	3.78 (3.75 to 3.81)	18.04 (17.95 to 18.13)	–19.43 (–19.55 to –19.3)	–$26,740	0.721

The results are based on 1000 simulation runs with a time horizon of 20 years in two hypothetical cohorts of 10,000 individuals (males and females respectively) with ages 40–69 yrs at the start of the simulation. The status quo is based on 17% coverage, 30% adherence, and the NPCDCS medication guideline. ^ Negative values indicate cost-saving, i.e., lower expenditure compared to status quo, and the values are calculated based on the estimated Indian population of age 40–69 yrs in 2020. ψ The estimated population of age 40–69 yrs in 2020 was used to calculate the annual expenditure for the population. # The probability of cost-saving was calculated based on the number of simulations runs which saved overall costs among the 1000 simulation runs.

### Budgetary implications of implementing the scenarios

The annual health expenditure on combined antihypertensive and CVD treatment reduced by $26.74 million ($26.74 mn) for the 70% scenario. We observed that an additional investment of $347.2 mn on hypertension control intervention resulted in a saving of $374 mn in CVD treatment expenditure.

### Increasing Coverage vis-à-vis Adherence

**Figure 2:**
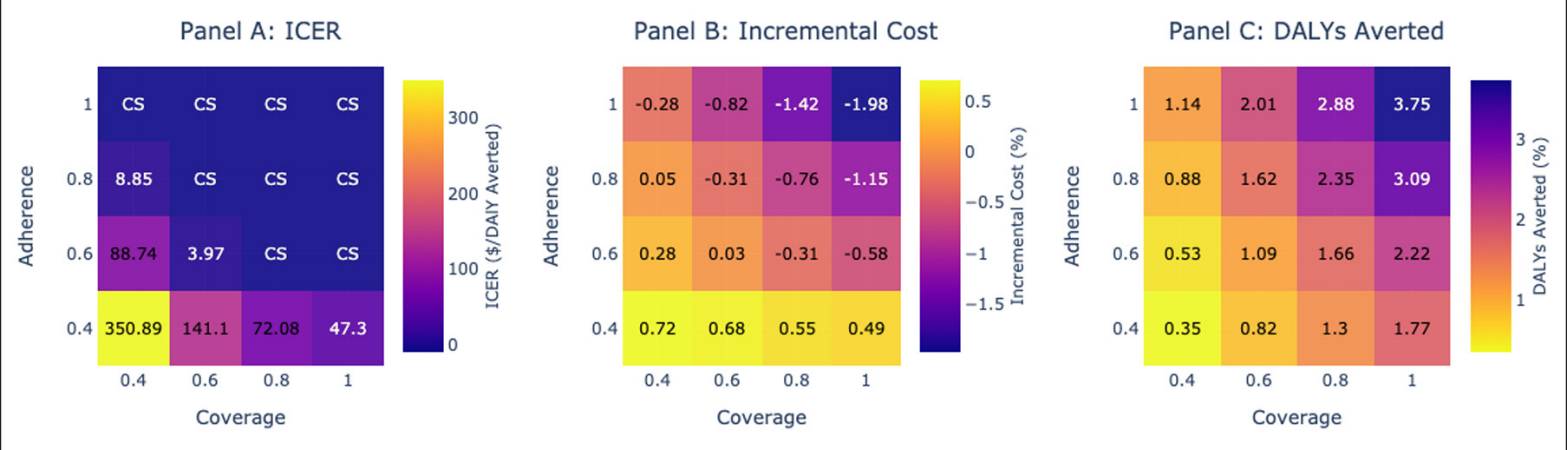
Increasing coverage vis-à-vis adherence. We assessed multiple scenarios of coverage and adherence to antihypertensive treatment compared to the status quo (coverage = 17%, adherence = 30%), and present the cost-effectiveness (panel A), incremental cost (panel B) and DALYs averted (panel C). Each column provides information for a coverage scenario with varying adherence, and each row provides information for a adherence scenario with varying treatment coverage. In panel A, the cells in blue indicate a cost-saving scenario, and cells in purple and yellow indicate a highly cost-effective scenario. The gradient of cell color is indicative of the changing cost-effectiveness. In panel B and C, the incremental cost and DALYs averted is presented with different scenarios of coverage and adherence compared to status quo. In general, moving from yellow to blue is advantageous. The results are based on 1000 simulation runs with a time horizon of 20 years in two hypothetical cohort of 10,000 individuals (males and females respectively) with ages of 40–69 yrs at the start of the simulation. ICER = Incremental Cost Effectiveness Ratio, DALY = Disability Adjusted Life Year, CS = Cost Saving.

In Figure 2, we plot the ICERs, incremental cost, and DALYs averted compared to status quo for varying coverage and adherence levels. An increase of coverage and adherence to 60% was almost budget neutral with an ICER of $3.97, and incremental cost between –0.01% and 0.06%. None of the scenarios with 40% treatment adherence was cost-saving. However, at 40% coverage, a cost-saving scenario could be achieved at 100% treatment adherence.

We also found that increasing adherence was more beneficial in improving cost savings. For example, increasing adherence to 80% while keeping coverage at 40% increased cost by 0.05% whereas increasing coverage to 80% while keeping adherence at 40% increased cost by 0.48%. However, 0.88% DALYs were averted in the former instance whereas 1.3% DALYs were averted in the latter instance.

### One-way Sensitivity Analysis

**Figure 3:**
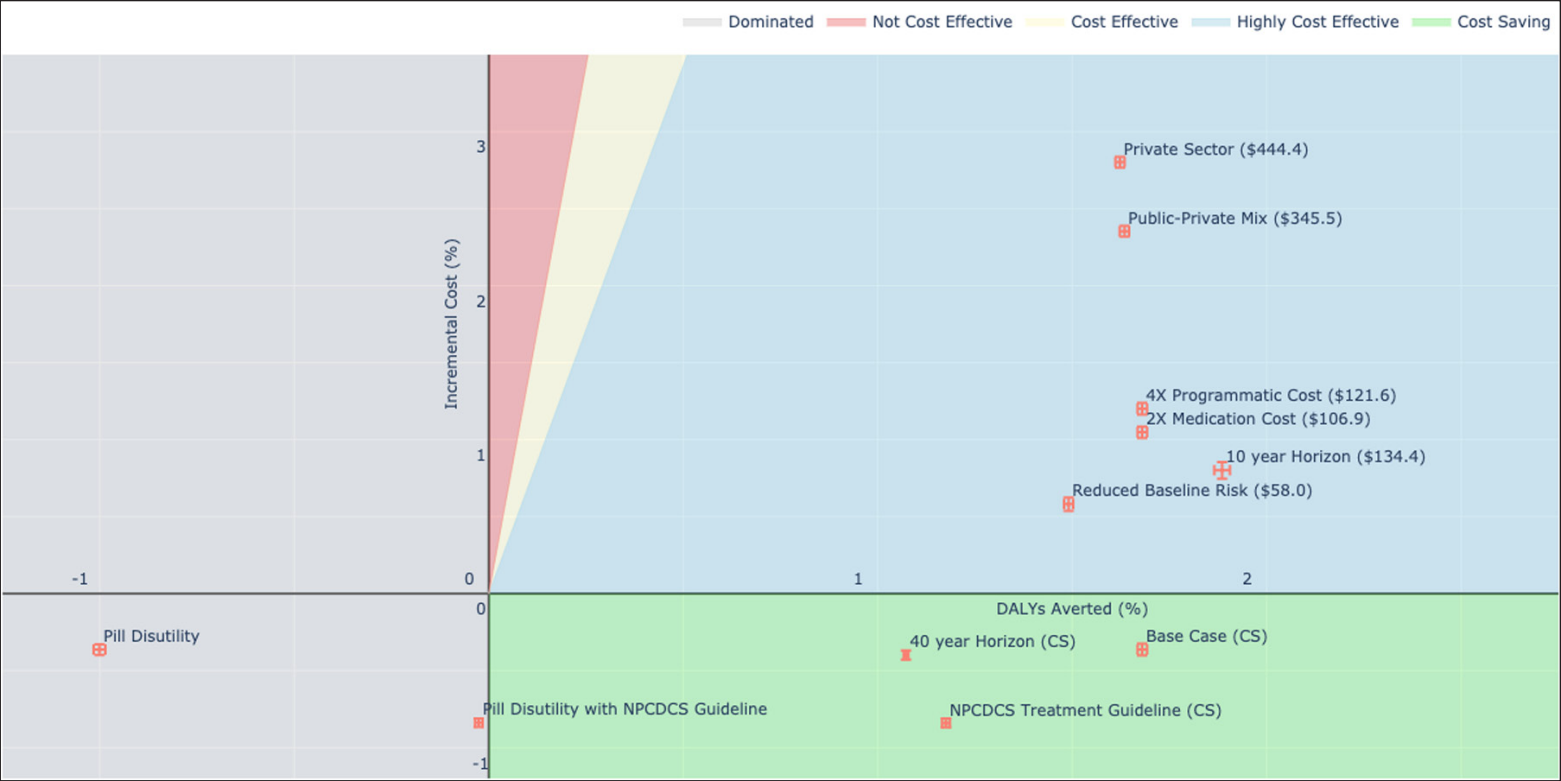
Incremental costs and DALYs averted under changes in sensitivity parameters. We assessed the robustness of our model through changes in select input parameters in a one-way sensitivity analysis. The x-axis and y-axis represent the DALYs averted and incremental cost compared to the status quo, and the error bars are based on the 95% confidence intervals. The text in the parenthesis along with the scenario name is the ICER ($/DALYs averted), with CS representing cost-saving scenario. The base case analysis) was cost-saving. If the medication cost was increased, the scenarios were no longer cost-saving, but the ICERs were below $110 and thus highly cost-effective. When the programmatic cost was quadrupled, the scenario did not save overall costs, but remained highly cost-effective. Under the assumption that the Globorisk calculator overestimates CVD risk, we ran the simulation with a 20% reduction in baseline risk and found the scenario no longer saved costs. If the treatment protocol was changed to the current NPCDCS guideline the cost savings increased. If the simulations were run with a reduced time horizon of 10 years, the scenario was highly cost-effective. When the simulations were run for a longer time-horizon of 40 years, the cost-savings increased. The values in the green band indicate cost savings, whereas the values in blue band indicate high cost-effectiveness. The results are based on 1000 simulation runs in two hypothetical cohort of 10,000 individuals (males and females respectively) with ages of 40–69 yrs at the start of simulation, with a high cost-effectiveness threshold of 0.5* GDP ($1169). ICER = Incremental Cost Effectiveness Ratio, GDP = Gross Domestic Product.

In one-way sensitivity analysis for the 70% scenario, the ICER increased to $120 for females and $90 for males when the antihypertensive medication cost was doubled. Also, when the programmatic cost was quadrupled, the ICER increased to $101 and $149 for females and males, respectively. The scenario was not cost-saving for the entire population with either of the changes. We observed an increase in ICER to $59 and $57 for females and males respectively when the baseline CVD risk was reduced by 20%. If we adopted the current NPCDCS treatment guideline, the scenario was cost-saving among both females and males. However, the health benefits were lower; 4.4% and 2.9% events averted among females and males respectively compared to 5.9% and 3.8% in the simplified protocol. If we consider the intervention with public-private mix, it is highly cost-effective with an ICER of $421 for females and $247 for males. With a reduced time-horizon of 10 years, the scenario remained highly cost-effective with ICERs of $134 for the entire population ($142 for females and $125 for males). If the time horizon was increased to 40 years, the intervention remains cost-saving among both females and males. The inclusion of disutility for being on chronic medication presented higher DALYs compared to status quo in 96% of the simulations. When the NPCDCS guideline was adopted with medication disutility, 54% of the simulations showed an increase in DALYs. We, thus, did not calculate the ICER for the scenarios. We have presented the incremental cost and DALYS averted along with ICER for each one-way sensitivity analysis in Figure 3.

## Discussion

Hypertension, the single largest preventable risk factor for CVDs, has a control rate of only 14% in India. Due to constraints on healthcare funding, a national hypertension control intervention is more likely to be scaled up if it is cost-saving. We find that such an intervention in India with a target of 70% coverage and adherence would be cost-saving and will save $25.6 mn annually on CVD prevention and treatment. There is an increase in savings as the coverage and adherence increases, with 100% coverage and adherence to antihypertensive treatment, potentially saving $145.5 mn annually. We also observe that at an adherence and coverage of 60%, the intervention starts being budget neutral. If an intervention needs to focus on either coverage or adherence, increasing adherence improves cost savings as it imparts higher benefit of the medication. However, a higher health impact is achieved by increasing coverage compared to adherence, since a larger proportion of hypertensive population is brought under treatment.

Previous studies have estimated that 3.8% of the non-communicable disease deaths in South Asia can be averted through increased coverage of antihypertensive treatment and the chronic diseases death rate can be reduced by 2% per annum by scaling treatment coverage to 70% [6,14]. We quantified the required economic resources and projected the health gains and cost savings for an intervention based on the tenets of IHCI, a national scale hypertension control program being implemented in India. Our study illustrates the effect of increasing coverage and adherence and enhances the understanding of previous studies on government-run insurance programs [51,58], scaling treatment coverage [59], increased opportunistic screening [60,61], and improved adherence [41]. The insights from our results are likely to hold for other LMICs with similar healthcare sector characteristics (growing disease burden of non-communicable diseases, low per-capita expenditure on primary care, and spending constraints of the respective governments on healthcare) [4,62]. However, the cost of hypertensive medication is low in India, and cost-effectiveness studies from other developing nations highlight the need to keep medication cost low for a successful scale up of hypertension control programs [63,64].

A previous study on government-run primary care insurance in India estimated an ICER of $469 per DALY averted at 34% treatment coverage and the NPCDCS treatment guideline [51]. In our model, the use of NPCDSC guidelines increased cost savings but averted fewer DALYs compared to the simplified treatment protocol. There is growing evidence that simpler, easy-to-follow treatment protocols can increase treatment coverage and improve downstream health outcomes [9,12,39]. Use of a simplified treatment protocol results in low CVD risk patients being initiated on treatment, and thus an increase in the overall expenditure on medication. However, inexpensive and generic version of the IHCI treatment protocol’s first-line drug, amlodipine, is widely available in the country. It is known to be safe and rarely leads to discontinuation due to adverse events [39]. Thus, treatment persistence is expected to be higher among individuals initiated on treatment and adverse event costs minimal.

With increased coverage, a larger proportion of hypertensive individuals will incur disutility due to the need to take daily medications for their remaining lifetimes. The disutility is due to the need of regular medication, and is a cause for patients to drop out of treatment [48,65]. In our sensitivity analysis, we find an increase in DALYs while increasing coverage for both the simplified and NPCDCS protocol. Previous cost-effectiveness studies have also shown that medication disutility exceeds the health gains when coverage of treatment is increased [58,66]. Though the concept of providing care based on an individual’s medication disutility is at its infancy in LMICs, treatment persistence could be improved if the patient-physician interaction includes exploration and addressal of the disutility.

In our study, we have not explored possible mechanisms to achieve increased coverage and adherence, and the additional programmatic cost associated with it. Though recent hypertension control pilots such as IHCI and mPower Heart project provide possible models of implementation, they currently do not serve patients in the private sector [11,17]. Our sensitivity analysis establishes that if the intervention paid for both public and private sector medication cost, the intervention will still be highly cost-effective. A comprehensive public health hypertension control program extended to both public and private sector will ensure that effective treatment can be availed by patients irrespective of their choice of providers.

The design of such a program can be further facilitated by frameworks and strategies outlined by WHO and World Heart Federation. WHO Package of Essential Noncommunicable (PEN) Disease Interventions can help administrators identify gaps in service delivery and improve health workforce training [67]. One of PEN’s components, the HEARTS technical package, was adopted in the implementation of IHCI [68]. The roadmap set by the World Heart Federation to reduce premature mortality due to CVDs by 25% by 2025 also helps identify and bridge the gaps for health systems to achieve the theoretical efficacy of the programs [69]. The roadmap helps identify the requirements from a health system to achieve the objectives. The mPower Heart project adopted the roadmap’s advocacy of task-shifting, decision-support systems, and improved tracking [17]. Further, while our study focusses on hypertension management, the frameworks set by WHO PEN and World Heart Federation can also help in designing preventive measures such as tobacco cessation and lifestyle improvements.

Our study is timely since several LMICs have recently initiated steps towards universal health coverage [70]. Their success will critically depend on the appropriate allocation of funds between preventive and curative services [51]. Under the Government of India’s national health insurance program, Ayushman Bharat [71], budgeted expenditure for hospitalization costs is thrice than that budgeted for the upgradation of health and wellness centres [71,72,73]. Increased investment in preventive programs such as the one studied here is necessary to ultimately reduce secondary and tertiary care expenditure, especially with the rapidly growing disease burden [4]. Also, such a preventive and primary healthcare program will further lower healthcare inequity and reduce out-of-pocket expenditure for households [21,74].

An implementation of such a large-scale intervention will not only need increased governmental support and commitment but also inter-departmental collaboration as expenditure on antihypertensive treatment and accrual of monetary benefits from CVD prevention will likely affect the budgets of different departments [9]. Although the intervention is cost-saving over 20 years, it will need a significant upfront investment. For instance, at 70% coverage and adherence, an annual expenditure of $347 mn in primary care would be needed, which represents 4% increase in India’s current health budget and is 14 times the current allocation for NPCDCS [75]. Though the required increase might appear daunting compared to the current allocation, it is important to note that India’s public health budget, at 1.28% of the GDP, is criticized for being low compared to the country’s needs and economic growth [14,76,77].

Our analysis has limitations. Our model assumes that changes in coverage and adherence are instantaneous although they may take up to five years in practice [14]. Although the programmatic cost during such a ramp-up period will be higher than that during steady-state, we expect our results to hold since the scenarios continued to be highly cost-effective even with quadrupled programmatic costs. Due to a lack of data from the Indian context, we were unable to model serious adverse events such as hypotension and electrolyte abnormality caused by antihypertensive treatment [78,79]. But their inclusion is unlikely to affect our results substantively as, firstly, less than 1% of hypertensive patients experience serious adverse events, and, secondly, we find that the 70% scenario is not highly cost-effective only if expenditure due to serious adverse events increases the cost incurred by more than 11.8% for every individual in the population. We did not capture the costs and disability due to other hypertension-related diseases, such as chronic kidney disease, due to lack of India-specific data but their inclusion will make the intervention scenarios more cost-saving than currently estimated. In terms of the cost-effectiveness thresholds, while we recognize that an opportunity-cost-based threshold would be more appropriate, we followed the per-capita GDP threshold due to a lack of consensus on the methods and data for the former [56].

Our model parameters are reliant on the GBD dataset by the Institute of Health Metrics and Evaluation. Though the GBD estimates have faced recent criticism from WHO member states, we believe the estimates are more transparent in terms of data availability and free from political pressures than other available estimates [80,81]. Further, we utilize the mortality and incidence estimates from the GBD dataset whereas most of the criticism is directed towards the estimates of DALYs. Also, we report the 95% uncertainty intervals for DALYs averted in our results (Table 2, Figure 3, and the detailed results in the supplementary appendix), and it accounts for some of the variability due to bias in GBD estimates of disease burden.

## Conclusion

The mathematic modeling study, utilizing India-specific data sources, found that scaling up antihypertensive treatment to cover 70% of the hypertensive individuals will save overall costs on CVD prevention and treatment. Achieving the cost-saving or budget-neutral scenario will require high levels of both coverage and adherence, which in turn, will require a sizeable investment in the short-term and will yield a reduction in expenditure in the long term.

## Data Accessibility Statements

Data and materials created as part of the study have been made publicly available and can be accessed at persistent doi 10.5281/zenodo.4046041. The WHO SAGE Wave 1 dataset and the Globorisk calculator were received upon request from the respective authors and have not been shared in the online repository. The detailed method, input parameters, and simulation results are provided in the Supplementary Appendix.

## Additional File

The additional file for this article can be found as follows:

10.5334/gh.952.s1 Web appendix. Cost effectiveness of improved hypertension management in India through increased treatment coverage and adherence: a mathematical modelling study.
