# Supplementary material for: Cost-Effectiveness of Improved Hypertension Management in India through Increased Treatment Coverage and Adherence: A Mathematical Modeling Study
Source: Glob Heart. 2021 May 10;16(1):37. doi: 10.5334/gh.952 (PMC8121007; doi:10.5334/gh.952)
Supplement: Web appendix. — Cost effectiveness of improved hypertension management in India through increased treatment coverage and adherence: a mathematical modelling study. [file gh-16-1-952-s1.pdf]

WEBAPPENDIX

Cost effectiveness of improved hypertension management in India through increased treatment coverage and adherence: a mathematical modelling study

Technical Details
- Microsimulation Model
- Probability of Clinical Events
- Modelling the coverage of hypertension treatment
- Modelling the treatment adherence to hypertension treatment
- Costs

Results
- Model Validation
- Cost and health outcomes for Overall Population
- Health outcomes for Females
- Health outcomes for Males
- One-way sensitivity analysis

Reporting Checklists
- Consolidated Health Economic Evaluation Reporting Standards (CHEERS) checklist

Tables

Webappendix Table S1: Illustration of current and next health states in the model
Webappendix Table S2: Age and sex-specific monthly probability of non-CVD death
Webappendix Table S3: Age and sex-specific probability of MI and Stroke upon CVD event
Webappendix Table S4: Age and sex-specific thirty-day probability of death after myocardial infarction and stroke
Webappendix Table S5: Age and sex-specific monthly probability of death for non-fatal myocardial infarction and stroke
Webappendix Table S6: Implementation of the treatment protocol
Webappendix Table S7: Age and SBP-specific relative risk of MI and Stroke due to antihypertensive medication
Webappendix Table S8: Cost of anti-hypertensive medication disaggregated by public sector and retail prices
Webappendix Table S9: Cost of CVD care medication disaggregated by public sector and retail prices
Webappendix Table S10: Programmatic cost of an IHCI-like program
Webappendix Table S11: Costs and health outcomes associated with the population of 40 to 69yr from 2020-40 across different coverage and adherence scenarios
Webappendix Table S12: Simulated health outcomes for Females between 40-69yr across different coverage and adherence scenarios
Webappendix Table S13: Simulated health outcomes for Males between 40-69yr across different coverage and adherence scenarios
Webappendix Table S14: One-way sensitivity analysis for costs and health outcomes associated with the population of 40 to 69yr from 2020-40 for the 70% coverage and adherence scenario
Webappendix Table S15: Simulated health outcomes for Females between 40-69yr across one-way sensitivity analysis
Webappendix Table S16: Simulated health outcomes for Males between 40-69yr across one-way sensitivity analysis
Webappendix Table S17: Consolidated Health Economic Evaluation Reporting Standards (CHEERS) checklist

Figures

Webappendix Figure S1: Schematic of Model Structure
Webappendix Figure S2: Validation of model output versus GBD 2017 estimates

Microsimulation Model

We developed a discrete-time microsimulation model to simulate individual progression through health states for a hypothetical cohort comprising of 10,000 individuals between 40 to 69 year old without history of CVD event (*Well* state) at the start of the simulation. The model follows each individual in one-month cycles for a simulation period of 20 years.

We simulated the model for two cohorts of 10,000 individuals each for males and females, respectively, with individual profiles sampled from the WHO SAGE Wave 1 dataset for India.[1] We ensured (i) the proportion of individuals in age brackets of 40-44, 45-49, 50-54, 55-59, 60-64, 65-69 in our simulated cohort was equal to the observed demographic characteristics for India, and (ii) the prevalence of hypertension in age- and sex-specific brackets in our simulated cohort was equal to the original dataset. We did not add individuals turning 40 years old in the subsequent cycles into the cohort.

In each cycle, an individual in the *Well* state can experience one of three possible clinical events and associated state transition: (i) no CVD event and thus stays in the current *Well* state (ii) occurrence of a CVD event and transitions to the *CVD Event* state, (iii) non-CVD related death and transitions to the terminal *Death* state.

The *CVD event* state comprises of an occurrence of either myocardial infarction (MI) or stroke. Upon entering the transitory *CVD Event* state, an individual experiences two possible clinical events: (i) a fatal CVD event, and thus moves to the terminal *Death* state, or (ii) a non-fatal CVD event and moves to either the *postMI* or *postStroke* chronic state. An individual in the *postMI* or *postStroke* state can experience one of three clinical events: (i) recurrence of a CVD event, and thus transition to *CVD Event* state, (ii) death and thus, move to the terminal *Death* state, or (iii) no CVD event, and stay in the current *postMI* or *postStroke* state. The structure of the model is provided in figure S1, along with the state transitions in table S1.

Probability of Clinical Events

Risk of a non-CVD Death

We retrieved the probability of all-cause deaths for India from WHO's Global Heath Data repository.[2] Further, we calculated the proportion of non-CVD deaths to all-cause deaths from GBD 2017 across 2012-17.[3] We were thus able to determine the monthly probability of non-CVD deaths in India (Table S2).

Risk of a CVD Event

We determined the risk of a CVD event using the Globorisk office calculator.[4] The inputs for the calculator include an individual's age, sex, systolic blood pressure, smoking habit and BMI. These values are available from the WHO SAGE Wave I dataset. The calculator provides 10-yr probability of a CVD event, and we converted it to a monthly probability assuming a constant hazard over the 10-year period.[5] We calculated the risk of a CVD event for each individual in the model every five years to incorporate the effect of increase in age and systolic blood pressure. The age-related increase in systolic blood pressure has been modelled based on a study by Bellows et al.[6] We calculated the conditional probability of either MI or stroke upon a CVD event (Table S3) based on the observed ratio of incidence of MI to stroke in the GBD 2017 study across 2012-17.[3]

Risk of death post CVD event

A CVD event could lead to either fatality within the first month (fatal CVD event), or the individual surviving for the first month (non-fatal CVD event). In case of a non-fatal CVD event, the individual experiences a higher risk of death compared to the risk of all-cause death among individuals in *Well* state. We calibrated the values for risk of death post MI or stroke such that the model estimated mortality matches the observed mortality of death in 2017 from the GBD 2017 study. The model validation result is presented in Figure S2, and the calibrated monthly probability of death due to MI and stroke in Table S4 and S5.

Risk of an additional CVD event

The monthly probability of a CVD event during the first month after an MI is based on the control arm in ACS QUIK study in Kerala, India.[7] The 30-day probability of repeat stroke during the first month is determined from the findings of Petty et al.[8] If an individual experiences a non-fatal MI or Stroke, the risk of an additional CVD event is based on a study by Lin et al.[9] The relative risk of death after an additional CVD event is 1.5 compared to risk of death after the first CVD event.[10]

Relative risk of CVD due to treatment

We determined the required drug dosage for which the individual's blood pressure decreases below 140mmHg based on the study by Law et al.[11] Based on the required dosage, we assigned specific combination of drugs for each individual based on the treatment protocol (Table S6). The treatment protocol is based on the approved

IHCI protocol being implemented in the state of Punjab currently.[12] Based on the combination of drugs administered, the initial blood pressure and the age of the individual, we determined the relative risk of MI and stroke from the study by Law et al (Table S7).

Modelling Coverage of Hypertension Treatment

The coverage of hypertension program was calculated as the ratio of hypertensive individuals on treatment to the total number of hypertensive individuals. We modeled the coverage of treatment by modifying two components: (i) the proportion of hypertensive individuals who were aware of their blood pressure, and (ii) the proportion of status-aware individuals initiated on treatment. To implement the first component, in each simulation run, we allocated individuals to be either aware of their blood pressure or not. The proportion of status-aware individuals in status-quo (i.e. current coverage of treatment in India) was based on findings of the Prospective Urban Rural Epidemiology Study (PURE) study.[13] For the second component, we first determined if the individuals, who were aware of their blood pressure, were also eligible for hypertensive treatment based on either the NPCDCS guideline (in status quo), or the simplified protocol (in intervention arm).[14,15] Further, among the eligible individuals, only a proportion of individuals were initiated on treatment. The treatment initiation ratio for the status quo was also based on the PURE study.[13] Based on the two components, we find 17% of the population to be covered by hypertension treatment in the status-quo, and we apply CVD risk reduction only to individuals who are initiated on the treatment.

Modelling Adherence to Hypertension Treatment

The adherence to treatment is calculated as the ratio of hypertensive individuals highly adherent to medication to the total number of individuals initiated on treatment. We modeled the adherence to treatment by modifying two components: (i) the proportion of individuals who persist with treatment for more than a year, (ii) the proportion of individuals who comply with the prescribed medication dosage and interval over the duration of their treatment. We implement the first component by assigning each individual either as persistent or non-persistent (discontinues treatment after an year). We used the results from a study by Van Wijk et al[16] for proportion of individuals persistent with treatment due to paucity of India-specific data. We assume that individuals who persist with treatment after one year neither drop out of treatment nor consume less than 50% of the prescribed medication.[16] In order to implement medication compliance among persistent individuals, we assign individuals to be either highly adherent or moderately adherent. Highly adherent individuals consume more than 80% of the pills on time and according to prescribed dosage. Moderately adherent individuals

consume between 50 to 80% of the pills on time and according to prescribed dosage. The proportion of highly adherent to medication is based on a study in India by Dennis et al.[17] The individuals who are highly adherent to medication experience the complete effect of the risk reduction, whereas individuals who are moderately adherent to medication experience only 50% of the risk reduction due to medication. This is based on a previous modelling study by Cherry et al.[18] Individuals who discontinue treatment after a year do not experience any reduction in risk of CVD.

Costs

We used data from the government contracts in the state of Telangana to determine the cost of antihypertensive drugs in the government sector.[19] We assumed a 30% overhead cost on the contract rates to account for logistics and inventory management of the drug.[20] The costs of drugs in the private sector was calculated by taking the mean selling price of the first five brand of drugs suggested by the Indian online pharmacy, 1mg.com.[21] The medication cost per individual was calculated by adding the cost of specific drug molecules in the individual's treatment regimen and their dosage for the individual. The costs have been provided in Table S8. For individuals moderately adherent on medication, we varied the costs between 50% and 80% (based on a normal distribution) of the fully adherent medication cost.

We assumed monthly doctor consultations for the first six months of treatment, followed by a quarterly consultation. The cost of doctor consultation was taken from a study by Prinja et al on community health centres and primary health centres across four states in India.[22] We included a one-time diagnostic test cost based on central government health scheme rate card.[23] The programmatic cost of a hypothetical intervention based on the tenets of IHCI was determined through discussions with experts and has been expanded in Table S10. The programmatic costs include the salaries of additional human resources, the cost of training the medical staff, and the operating costs for running the tech platform. We the required resources and their respective cost was based on IHCI's implementation in nine districts of Telangana.

The cost of acute CVD care comprised of consultation, hospital room costs, procedures, and medication cost and the cost of chronic CVD care comprised of outpatient consultation cost and chronic medication cost. The values were based on data from WHO-CHOICE,[24] and calculated by a previous cost-effectiveness study by Lin et al.[9] Further, we included the cost of percutaneous coronary intervention (PCI) and coronary artery bypass graft(CABG) among 4.2% and 1.7% of the patients with acute MI.[25] All individuals with chronic

CVD incurred CVD medication cost. The cost of chronic CVD medication in the government sector was determined from the median buyer price in the International Drug Price Indicator.[26] The cost of CVD medication cost in private sector was based on the mean selling price of the first five brands suggested by 1mg.com.[21] The costs have been provided in Table S9. All costs were scaled to the year 2020 with an inflation factor of 3% per annum.

Webappendix Table S1: Illustration of current and next health states in the model

#	Current State	Possible Next State(s)
1	Well	Well
		CVD Event
		Death
2	CVD Event	postMI
		postStroke
		Death
3	postMI	postMI
		CVD Event
		Death
4	postStroke	postStroke
		CVD Event
		Death
5	Death	Death

Webappendix Table S2: Age and sex-specific monthly probability of non-CVD death

Age	Female*	Male*
40-44	0.000206-0.000210	0.000324-0.000330
45-49	0.000257-0.000263	0.000427-0.000434
50-54	0.000381-0.000389	0.000568-0.000579
55-59	0.000544-0.000554	0.000834-0.000849
60-64	0.001013-0.001026	0.001224-0.001245
65-69	0.001658-0.001679	0.002029-0.002058
70-74	0.002744-0.002773	0.003114-0.003153
75-79	0.004026-0.004072	0.004574-0.004626
80-84	0.006411-0.006512	0.006525-0.006648
85 +	0.011137-0.011312	0.011201-0.011413

* The ranges provided are 95% CI for monthly probability of death due to all causes except CVDs, with the values sampled based on a β distribution in the simulation runs.

Webappendix Table S3: Age and sex-specific proportion of MI to Stroke upon CVD event

Age	Female*	Male*
40-44	0.375-0.378	0.435-0.444
45-49	0.384-0.386	0.471-0.484
50-54	0.418-0.424	0.507-0.527
55-59	0.451-0.461	0.534-0.560
60-64	0.497-0.510	0.562-0.589
65-69	0.536-0.551	0.587-0.611
70-74	0.557-0.574	0.625-0.647
75-79	0.569-0.589	0.668-0.691
80-84	0.560-0.574	0.660-0.677
85 +	0.560-0.574	0.660-0.677

* The ranges provided are 95% CI for ratio of MI to Stroke occurrence upon a CVD event, with the values sampled based on a β distribution in the simulation runs.

Webappendix Table S4: Age and sex-specific thirty-day probability of death after myocardial infarction and stroke

Age	Female		Male	
	MI	Stroke	MI	Stroke
40-44	0.0132	0.1188	0.0330	0.1262
45-49	0.0132		0.0330	
50-54	0.0132		0.0330	
55-59	0.0188		0.0470	
60-64	0.0188		0.0470	
65-69	0.0356		0.0890	
70-74	0.0356		0.0890	
75-79	0.0516		0.1290	
80+	0.0516		0.1290	

Webappendix Table S5: Age and sex-specific monthly probability of death for non-fatal myocardial infarction and stroke

Age	Female		Male	
	MI	Stroke	MI	Stroke
40-44	0.000525	0.000541	0.00499	0.001699
45-49	0.001981	0.000916	0.005487	0.001699
50-54	0.002155	0.001456	0.005786	0.002398
55-59	0.002562	0.001622	0.006085	0.003196
60-64	0.002969	0.001747	0.006085	0.003894
65-69	0.003317	0.002120	0.006980	0.005288
70-74	0.003491	0.002494	0.008470	0.006582
75-79	0.003897	0.002991	0.009760	0.008371
80+	0.009041	0.004893	0.019342	0.012534

Webappendix Table S6: Implementation of the treatment protocol

Initial Systolic Blood Pressure (mmHg)	Medication Administered	Estimated reduction in systolic blood pressure (mmHg)
140	Amlodipine 5mg	5.9
150	Amlodipine 10mg	8.7
150	Amlodipine 10mg + Telmisartan 40mg	15.4
160	Amlodipine 10mg + Telmisartan 80mg	18.4
160	Amlodipine 10mg + Telmisartan 80mg + Chlorthalidone 12.5mg	25.9
170	Amlodipine 10mg + Telmisartan 80mg + Chlorthalidone 25mg	29
180	Amlodipine 10mg + Telmisartan 80mg + Chlorthalidone 25mg	31.7

Webappendix Table S7: Age and SBP-specific relative risk of MI and Stroke due to antihypertensive medication

Age	Initial SBP (in mmHg)	Relative Risk	
		MI	ST
40 - 49	140	0.810	0.740
	150	0.675	0.575
	160	0.485	0.355
	170	0.390	0.255
	180+	0.320	0.200
50 - 59	140	0.810	0.750
	150	0.685	0.595
	160	0.495	0.375
	170	0.400	0.275
	180+	0.330	0.220
60 - 69	140	0.830	0.780
	150	0.710	0.630
	160	0.535	0.425
	170	0.440	0.320
	180+	0.380	0.260
70 -79	140	0.860	0.890
	150	0.755	0.805
	160	0.595	0.665
	170	0.505	0.585
	180+	0.440	0.530
80 +	140	0.890	0.890
	150	0.805	0.805
	160	0.665	0.665
	170	0.585	0.585
	180+	0.530	0.530

Webappendix Table S8: Cost of anti-hypertensive medication disaggregated by public sector and retail prices

Drug	Cost per pill	
	Public Sector (INR)	Retail (INR)
Amlodipine 5mg	0.169	1.04
Telmisartan 40mg	0.767	2.72
Chlorthalidone 12.5mg	0.780	8.24

Webappendix Table S9: Cost of CVD care medication disaggregated by public sector and retail prices

Drug	Cost per pill	
	Public Sector (INR)	Retail (INR)
Aspirin 75 mg	0.98	0.28
Atenolol 50 mg	0.721	1.75
Lisinopril 10 mg	9.38	12.40
Simvastatin 20 mg	3.717	14.55

Webappendix Table S10: Programmatic cost of an IHCI-like program

**Parameter**		**Value**
Number of districts served		9
Population covered		9545455
Proportion of population greater than 40yr		27%
Population serviced by the intervention		2577273
**Cost Entities**	**Number of entities**	**Cost per entity (INR)**
Cardiovascular Health Officer	4	150,000 per month
State Treatment Supervisor	9	31,000 per month
Training sessions per district per annum	12	100,000 per session
Technology teams allocated to a state	1	100,000 per month
Platform infrastructure	1	100,000 per annum
Annual Cost of Intervention (INR)		2,26,48,000
Cost of Intervention per individual serviced (INR)		8.79
Cost of Intervention per individual serviced (US $)		0.13

Webappendix Table S11: Costs and health outcomes associated with the population of 40 to 69yr from 2020-40 across different coverage and adherence scenarios

Coverage	Adherence	ICER ($/DALY averted)	DALYs Averted (percent, 95% UI)	Incremental Cost (percent, 95% UI)	Per-capita incremental costs over 20 years: Antihypertensive Program ($, 95% UI)	Per-capita incremental costs over 20 years: CVD Treatment ($, 95% UI)	Annual net expenditure (in '000 US $)	Probability of Cost Saving (percent)
**0.4**	0.4	350.89	0.347 (0.342 to 0.353)	0.72 (0.7 to 0.73)	6.62 (6.59 to 6.65)	-3.88 (-3.94 to -3.83)	52649	0.004
**0.4**	0.6	88.74	0.528 (0.522 to 0.535)	0.28 (0.26 to 0.29)	7.09 (7.06 to 7.12)	-6.04 (-6.11 to -5.97)	20255	0.172
**0.4**	0.8	8.85	0.877 (0.869 to 0.885)	0.05 (0.02 to 0.07)	10.28 (10.23 to 10.33)	-10.11 (-10.19 to -10.03)	3354	0.451
**0.4**	1	Cost Saving	1.141 (1.132 to 1.15)	-0.28 (-0.3 to -0.25)	12.1 (12.04 to 12.16)	-13.16 (-13.25 to -13.07)	-20392	0.737
**0.6**	0.4	141.10	0.821 (0.811 to 0.83)	0.68 (0.65 to 0.71)	11.83 (11.77 to 11.9)	-9.23 (-9.34 to -9.13)	50020	0.078
**0.6**	0.6	3.97	1.091 (1.081 to 1.101)	0.03 (-0.01 to 0.06)	12.54 (12.48 to 12.61)	-12.45 (-12.56 to -12.34)	1870	0.478
**0.6**	0.8	Cost Saving	1.615 (1.603 to 1.627)	-0.31 (-0.35 to -0.27)	17.32 (17.23 to 17.41)	-18.51 (-18.63 to -18.39)	-22881	0.695
**0.6**	1	Cost Saving	2.015 (2.002 to 2.027)	-0.82 (-0.86 to -0.78)	20.05 (19.95 to 20.15)	-23.18 (-23.3 to -23.05)	-60101	0.897
**0.7**	0.7	Cost Saving	1.68 (1.668 to 1.692)	-0.36 (-0.4 to -0.33)	18.04 (17.95 to 18.13)	-19.43 (-19.55 to -19.3)	-26741	0.721
**0.8**	0.4	72.08	1.301 (1.29 to 1.313)	0.55 (0.51 to 0.59)	17.04 (16.95 to 17.13)	-14.94 (-15.05 to -14.82)	40519	0.174
**0.8**	0.6	Cost Saving	1.661 (1.649 to 1.673)	-0.31 (-0.35 to -0.27)	17.98 (17.89 to 18.08)	-19.17 (-19.29 to -19.04)	-22785	0.688
**0.8**	0.8	Cost Saving	2.352 (2.338 to 2.365)	-0.76 (-0.81 to -0.72)	24.34 (24.21 to 24.46)	-27.26 (-27.39 to -27.12)	-56155	0.857
**0.8**	1	Cost Saving	2.879 (2.865 to 2.894)	-1.42 (-1.47 to -1.37)	27.98 (27.84 to 28.12)	-33.4 (-33.54 to -33.26)	-104243	0.957
**1**	0.4	47.30	1.77 (1.758 to 1.782)	0.49 (0.45 to 0.53)	22.24 (22.12 to 22.37)	-20.36 (-20.49 to -20.24)	36166	0.256
**1**	0.6	Cost Saving	2.218 (2.205 to 2.231)	-0.58 (-0.63 to -0.54)	23.42 (23.3 to 23.54)	-25.65 (-25.79 to -25.51)	-42907	0.789
**1**	0.8	Cost Saving	3.086 (3.071 to 3.102)	-1.15 (-1.21 to -1.1)	31.36 (31.2 to 31.52)	-35.77 (-35.91 to -35.62)	-84846	0.914
**1**	1	Cost Saving	3.751 (3.734 to 3.768)	-1.98 (-2.04 to -1.92)	35.93 (35.75 to 36.11)	-43.5 (-43.65 to -43.34)	-145542	0.984

Webappendix Table S12: Simulated health outcomes for Females between 40-69yr across different coverage and adherence scenarios

Coverage	Adherence	Incremental Cost (percent, 95% UI)	DALYs Averted (percent, 95% UI)	CVD Events Averted (percent, 95% UI)	CVD Deaths Averted (percent, 95% UI)	ICER ($/DALY averted)
**0.4**	0.4	0.87 (0.84 to 0.9)	0.441 (0.431 to 0.451)	1.114 (1.096 to 1.131)	1.166 (1.135 to 1.198)	346.95
**0.4**	0.6	0.33 (0.3 to 0.36)	0.677 (0.666 to 0.688)	1.783 (1.762 to 1.803)	1.862 (1.827 to 1.897)	86.48
**0.4**	0.8	0.05 (0.01 to 0.08)	1.131 (1.118 to 1.145)	3.031 (3.006 to 3.056)	3.147 (3.107 to 3.186)	7.19
**0.4**	1	-0.35 (-0.39 to -0.31)	1.476 (1.462 to 1.491)	3.979 (3.952 to 4.007)	4.11 (4.066 to 4.154)	Cost Saving
**0.6**	0.4	0.77 (0.73 to 0.82)	1.053 (1.037 to 1.068)	2.775 (2.745 to 2.804)	2.894 (2.846 to 2.943)	128.72
**0.6**	0.6	-0.02 (-0.07 to 0.03)	1.409 (1.392 to 1.426)	3.778 (3.747 to 3.809)	3.917 (3.865 to 3.968)	Cost Saving
**0.6**	0.8	-0.43 (-0.48 to -0.37)	2.088 (2.069 to 2.106)	5.626 (5.593 to 5.659)	5.84 (5.785 to 5.895)	Cost Saving
**0.6**	1	-1.04 (-1.1 to -0.98)	2.607 (2.587 to 2.628)	7.055 (7.02 to 7.09)	7.323 (7.265 to 7.381)	Cost Saving
**0.7**	0.7	-0.46 (-0.51 to -0.4)	2.169 (2.15 to 2.188)	5.852 (5.817 to 5.887)	6.071 (6.013 to 6.129)	Cost Saving
**0.8**	0.4	0.64 (0.59 to 0.7)	1.676 (1.658 to 1.694)	4.46 (4.428 to 4.493)	4.639 (4.584 to 4.694)	67.34
**0.8**	0.6	-0.39 (-0.45 to -0.33)	2.148 (2.129 to 2.167)	5.778 (5.744 to 5.812)	5.99 (5.933 to 6.047)	Cost Saving
**0.8**	0.8	-0.96 (-1.02 to -0.89)	3.051 (3.029 to 3.072)	8.25 (8.214 to 8.287)	8.565 (8.504 to 8.626)	Cost Saving
**0.8**	1	-1.76 (-1.83 to -1.69)	3.741 (3.718 to 3.764)	10.158 (10.121 to 10.196)	10.531 (10.468 to 10.595)	Cost Saving
**1**	0.4	0.54 (0.48 to 0.61)	2.285 (2.265 to 2.305)	6.133 (6.099 to 6.168)	6.36 (6.301 to 6.42)	41.82
**1**	0.6	-0.76 (-0.82 to -0.69)	2.874 (2.852 to 2.895)	7.786 (7.748 to 7.824)	8.057 (7.994 to 8.12)	Cost Saving
**1**	0.8	-1.46 (-1.53 to -1.38)	4.005 (3.981 to 4.029)	10.869 (10.83 to 10.908)	11.257 (11.191 to 11.323)	Cost Saving
**1**	1	-2.46 (-2.54 to -2.38)	4.875 (4.849 to 4.901)	13.247 (13.207 to 13.286)	13.743 (13.674 to 13.811)	Cost Saving

Webappendix Table S13: Simulated health outcomes for Males between 40-69yr across different coverage and adherence scenarios

Coverage	Adherence	Incremental Cost (percent, 95% UI)	DALYs Averted (percent, 95% UI)	CVD Events Averted (percent, 95% UI)	CVD Deaths Averted (percent, 95% UI)	ICER ($/DALY averted)
**0.4**	0.4	0.59 (0.57 to 0.61)	0.276 (0.27 to 0.281)	0.705 (0.695 to 0.715)	0.705 (0.688 to 0.723)	354.59
**0.4**	0.6	0.23 (0.21 to 0.25)	0.413 (0.406 to 0.42)	1.138 (1.126 to 1.151)	1.12 (1.098 to 1.142)	91.58
**0.4**	0.8	0.05 (0.02 to 0.07)	0.681 (0.672 to 0.69)	1.942 (1.926 to 1.958)	1.919 (1.892 to 1.946)	10.97
**0.4**	1	-0.22 (-0.24 to -0.19)	0.882 (0.872 to 0.893)	2.553 (2.535 to 2.572)	2.526 (2.495 to 2.556)	Cost Saving
**0.6**	0.4	0.61 (0.57 to 0.64)	0.641 (0.63 to 0.653)	1.747 (1.723 to 1.77)	1.767 (1.731 to 1.803)	156.78
**0.6**	0.6	0.07 (0.03 to 0.1)	0.845 (0.833 to 0.858)	2.394 (2.369 to 2.419)	2.388 (2.35 to 2.426)	13.06
**0.6**	0.8	-0.21 (-0.26 to -0.17)	1.25 (1.236 to 1.264)	3.611 (3.584 to 3.638)	3.605 (3.563 to 3.648)	Cost Saving
**0.6**	1	-0.63 (-0.68 to -0.59)	1.557 (1.541 to 1.572)	4.542 (4.513 to 4.571)	4.513 (4.47 to 4.557)	Cost Saving
**0.7**	0.7	-0.29 (-0.33 to -0.24)	1.303 (1.289 to 1.316)	3.78 (3.752 to 3.808)	3.756 (3.714 to 3.799)	Cost Saving
**0.8**	0.4	0.48 (0.43 to 0.52)	1.012 (0.999 to 1.025)	2.875 (2.849 to 2.901)	2.882 (2.842 to 2.922)	78.13
**0.8**	0.6	-0.24 (-0.29 to -0.2)	1.284 (1.27 to 1.299)	3.739 (3.712 to 3.767)	3.713 (3.671 to 3.755)	Cost Saving
**0.8**	0.8	-0.6 (-0.65 to -0.55)	1.812 (1.797 to 1.827)	5.335 (5.305 to 5.364)	5.301 (5.255 to 5.346)	Cost Saving
**0.8**	1	-1.13 (-1.19 to -1.08)	2.214 (2.197 to 2.23)	6.557 (6.527 to 6.588)	6.5 (6.453 to 6.548)	Cost Saving
**1**	0.4	0.45 (0.4 to 0.5)	1.372 (1.359 to 1.386)	3.942 (3.916 to 3.969)	3.954 (3.912 to 3.996)	54.35
**1**	0.6	-0.44 (-0.49 to -0.39)	1.711 (1.696 to 1.726)	5.005 (4.978 to 5.033)	4.997 (4.952 to 5.042)	Cost Saving
**1**	0.8	-0.9 (-0.96 to -0.85)	2.377 (2.36 to 2.394)	7.023 (6.993 to 7.053)	6.986 (6.938 to 7.034)	Cost Saving
**1**	1	-1.58 (-1.64 to -1.52)	2.882 (2.864 to 2.901)	8.566 (8.534 to 8.598)	8.499 (8.45 to 8.548)	Cost Saving

Webappendix Table S14: One-way sensitivity analysis for costs and health outcomes associated with the population of 40 to 69yr from 2020-40 for the 70% coverage and adherence scenario

Sensitivity Scenario	ICER ($/DALY averted)	DALYs Averted (percent, 95% UI)	Per-capita incremental costs over 20 years		Annual net expenditure for 40-69yr population (in '000 US $)	Probability of Cost Saving
			Antihypertensive Program ($, 95% UI)	CVD Treatment ($, 95% UI)		
**Results using main input assumptions**	Cost Saving	1.68 (1.668 to 1.692)	-0.36 (-0.4 to -0.33)	18.04 (17.95 to 18.13)	-19.43 (-19.55 to -19.3)	0.721
**2X Antihypertensive Medication Cost**	106.92	1.68 (1.668 to 1.692)	1.05 (1.01 to 1.09)	23.44 (23.35 to 23.54)	-19.41 (-19.54 to -19.29)	0.039
**4X Programmatic Cost**	121.64	1.68 (1.668 to 1.692)	1.2 (1.16 to 1.24)	24.01 (23.92 to 24.11)	-19.43 (-19.55 to -19.3)	0.025
**20% Reduction in Baseline CVD Risk**	58.00	1.49 (1.479 to 1.502)	0.58 (0.54 to 0.62)	18.37 (18.28 to 18.46)	-16.55 (-16.66 to -16.43)	0.201
**NPCDCS Medication Guideline**	Cost Saving	1.175 (1.166 to 1.185)	-0.84 (-0.87 to -0.81)	10.76 (10.72 to 10.8)	-13.97 (-14.08 to -13.86)	0.967
**Only Private Sector**	444.38	1.623 (1.611 to 1.635)	2.8 (2.77 to 2.84)	44.33 (44.21 to 44.45)	-28.14 (-28.33 to -27.95)	0
**Public-Private Sector Mix**	345.46	1.634 (1.622 to 1.646)	2.35 (2.32 to 2.39)	39.07 (38.96 to 39.18)	-26.4 (-26.58 to -26.22)	0
**10 year Time Horizon**	134.38	1.885 (1.865 to 1.905)	0.8 (0.75 to 0.85)	11.54 (11.47 to 11.6)	-10.09 (-10.18 to -10.01)	0.192
**40 year Time Horizon**	Cost Saving	1.073 (1.067 to 1.079)	-0.4 (-0.43 to -0.37)	23.3 (23.19 to 23.41)	-25.56 (-25.71 to -25.41)	0.786

Webappendix Table S15: Simulated health outcomes for Females between 40-69yr across one-way sensitivity analysis

Sensitivity Scenario	Incremental Cost (percent, 95% UI)	DALYs Averted (percent, 95% UI)	CVD Events Averted (percent, 95% UI)	CVD Deaths Averted (percent, 95% UI)	ICER ($/DALY averted)
**Results using main input assumptions**	-0.46 (-0.51 to -0.4)	2.169 (2.15 to 2.188)	5.852 (5.817 to 5.887)	6.071 (6.013 to 6.129)	Cost Saving
**2X Antihypertensive Medication Cost**	1.47 (1.42 to 1.53)	2.169 (2.15 to 2.188)	5.852 (5.817 to 5.887)	6.071 (6.013 to 6.129)	120.18
**4X Programmatic Cost**	1.24 (1.19 to 1.3)	2.169 (2.15 to 2.188)	5.852 (5.817 to 5.887)	6.071 (6.013 to 6.129)	100.74
**20% Reduction in Baseline CVD Risk**	0.73 (0.67 to 0.8)	1.918 (1.899 to 1.937)	6.174 (6.135 to 6.214)	6.21 (6.146 to 6.274)	58.72
**NPCDCS Medication Guideline**	-1.24 (-1.29 to -1.2)	1.559 (1.542 to 1.575)	4.411 (4.38 to 4.442)	4.577 (4.525 to 4.628)	Cost Saving
**Only Private Sector**	4.24 (4.18 to 4.29)	2.094 (2.075 to 2.113)	5.559 (5.525 to 5.593)	5.752 (5.695 to 5.809)	540.39
**Public-Private Sector Mix**	3.57 (3.52 to 3.63)	2.109 (2.09 to 2.128)	5.617 (5.584 to 5.651)	5.816 (5.758 to 5.873)	421.64
**10 year Time Horizon**	1.07 (0.98 to 1.15)	2.452 (2.419 to 2.485)	6.871 (6.817 to 6.925)	6.967 (6.858 to 7.076)	142.15
**40 year Time Horizon**	-0.42 (-0.46 to -0.37)	1.331 (1.322 to 1.34)	4.278 (4.254 to 4.302)	4.454 (4.422 to 4.486)	Cost Saving

Webappendix Table S16: Simulated health outcomes for Males between 40-69yr across one-way sensitivity analysis

Sensitivity Scenario	Incremental Cost (percent, 95% UI)	DALYs Averted (percent, 95% UI)	CVD Events Averted (percent, 95% UI)	CVD Deaths Averted (percent, 95% UI)	ICER ($/DALY averted)
**Results using main input assumptions**	-0.29 (-0.33 to -0.24)	1.303 (1.289 to 1.316)	3.78 (3.752 to 3.808)	3.756 (3.714 to 3.799)	Cost Saving
**2X Antihypertensive Medication Cost**	0.7 (0.66 to 0.75)	1.303 (1.289 to 1.316)	3.78 (3.752 to 3.808)	3.756 (3.714 to 3.799)	89.89
**4X Programmatic Cost**	1.17 (1.12 to 1.21)	1.303 (1.289 to 1.316)	3.78 (3.752 to 3.808)	3.756 (3.714 to 3.799)	148.50
**20% Reduction in Baseline CVD Risk**	0.46 (0.41 to 0.51)	1.161 (1.148 to 1.174)	4.067 (4.037 to 4.097)	3.915 (3.868 to 3.963)	57.08
**NPCDCS Medication Guideline**	-0.51 (-0.54 to -0.48)	0.88 (0.868 to 0.891)	2.87 (2.845 to 2.895)	2.804 (2.766 to 2.841)	Cost Saving
**Only Private Sector**	1.62 (1.58 to 1.66)	1.259 (1.245 to 1.272)	3.595 (3.568 to 3.622)	3.553 (3.511 to 3.594)	321.09
**Public-Private Sector Mix**	1.35 (1.31 to 1.39)	1.267 (1.254 to 1.281)	3.632 (3.605 to 3.659)	3.594 (3.552 to 3.635)	247.61
**10 year Time Horizon**	0.6 (0.54 to 0.66)	1.472 (1.45 to 1.494)	4.319 (4.281 to 4.357)	4.291 (4.215 to 4.367)	124.96
**40 year Time Horizon**	-0.38 (-0.42 to -0.35)	0.854 (0.847 to 0.861)	3.319 (3.297 to 3.34)	3.261 (3.234 to 3.287)	Cost Saving

Webappendix Figure 1: Schematic of Model Structure

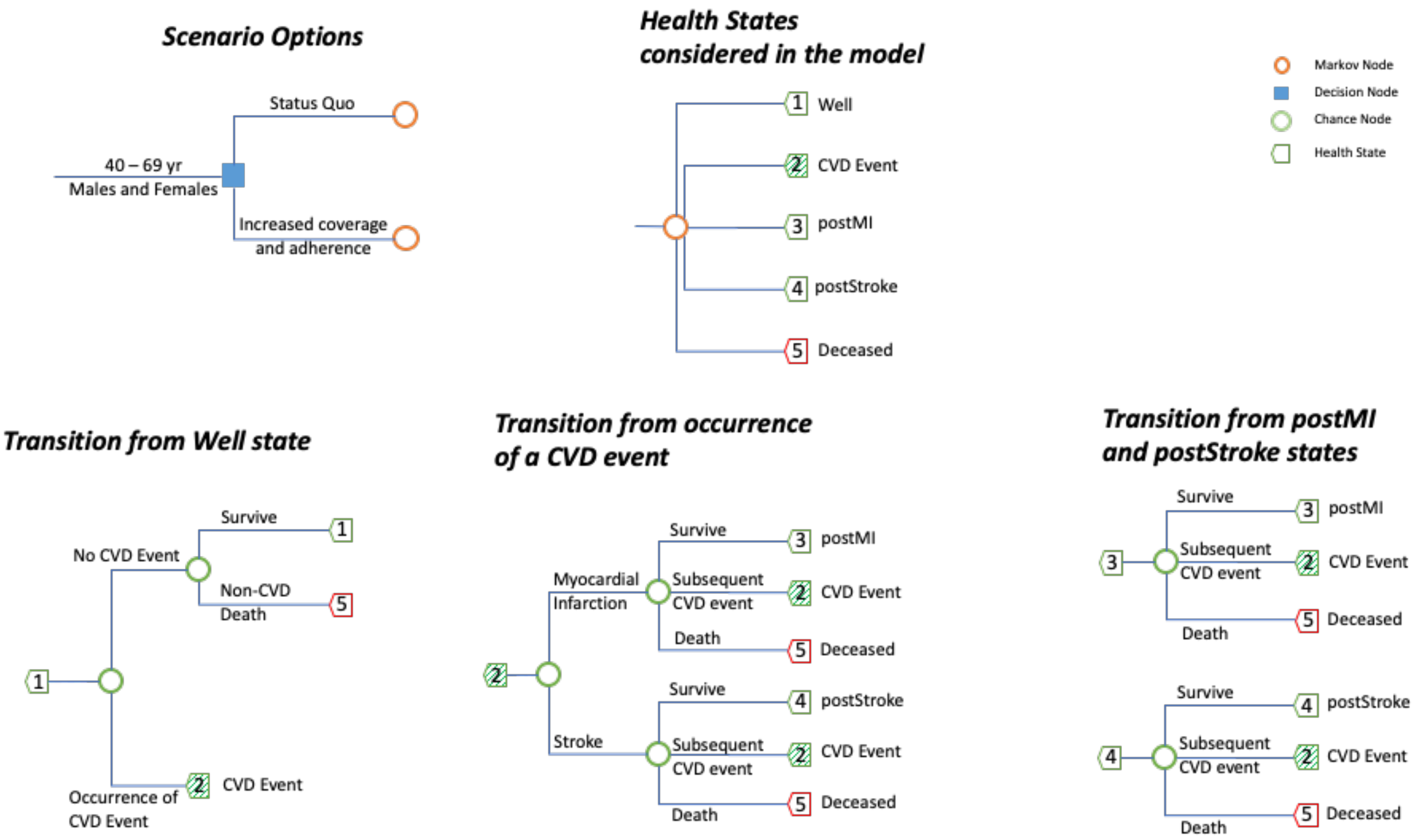

The blue square indicates the choice between various intervention scenarios, and the orange circle indicates the chosen intervention. The health states (indicated by green pentagon) comprises of (1) well (no past CVD event), (2) occurrence of a CVD event, (3) surviving post a myocardial infarction (postMI), (4) surviving post a stroke (postStroke), and (5) deceased state. The blue-colored branches from each heath state lead to another heath state based on the probability of the intermediate event (indicated by the green circle). The (2) CVD Event is a transitionary markov state and comprises of either an occurrence of MI or stroke.

Webappendix Figure 2: Validation of model output versus GBD 2017 estimates

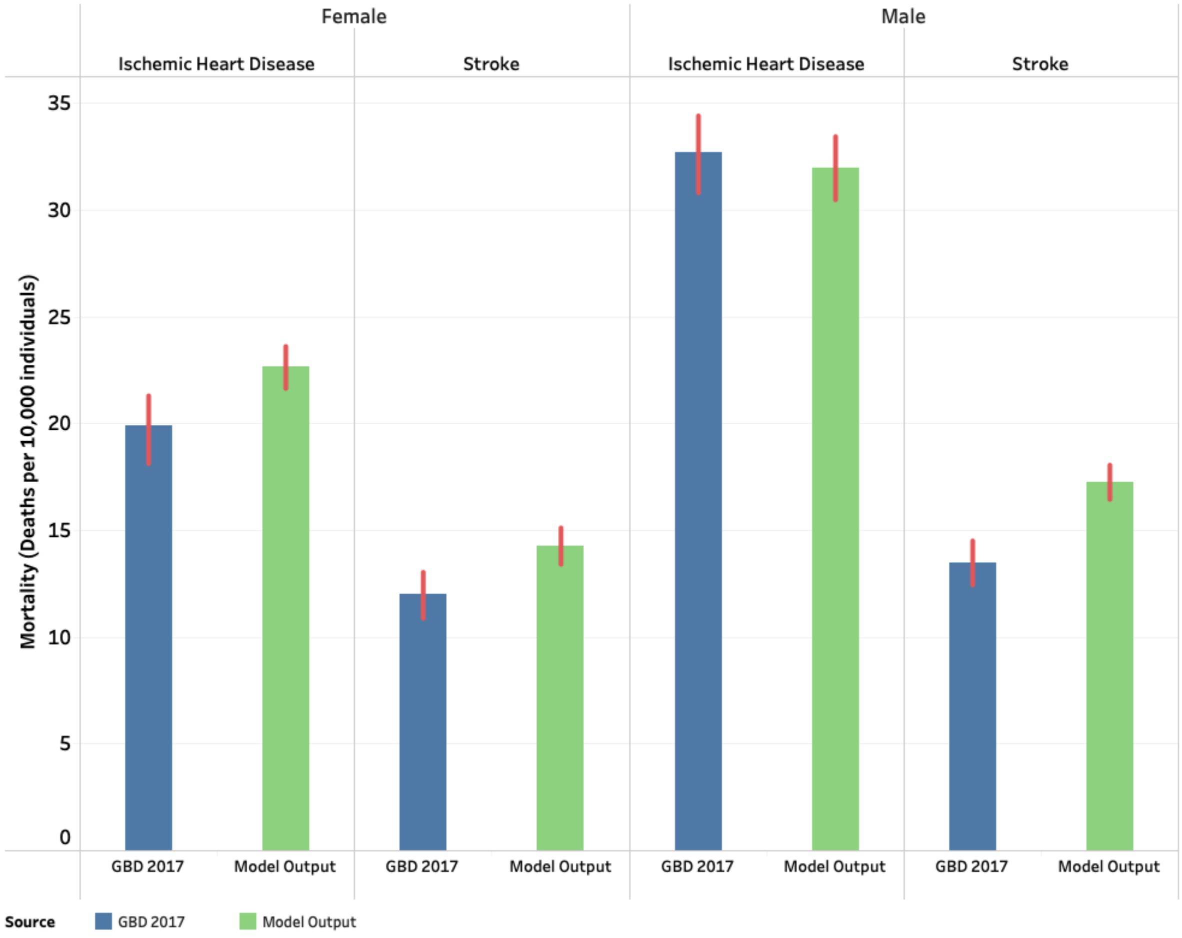

The figure provides the average deaths predicted by the microsimulation model on average over 5 years. We compare our predicted values with the Global Burden of Disease study's estimated deaths for 2017. The blue bar and green bar represent the mean deaths for GBD 2017 and model output respectively. The red line represents the 95% confidence intervals. The estimates have been disaggregated by sex and cause of death (Ischemic Heart Disease or Stroke).

Webappendix Table 17: Consolidated Health Economic Evaluation Reporting Standards (CHEERS) checklist[27]

Section/item	Item No	Recommendation	Reported on page No/ line No
**Title and abstract**			
**Title**	1	Identify the study as an economic evaluation or use more specific terms such as "cost-effectiveness analysis", and describe the interventions compared.	Page# 1
**Abstract**	2	Provide a structured summary of objectives, perspective, setting, methods (including study design and inputs), results (including base case and uncertainty analyses), and conclusions.	Page# 2
**Introduction**			
**Background and objectives**	3	Provide an explicit statement of the broader context for the study.	10-16
		Present the study question and its relevance for health policy or practice decisions.	35-40
**Methods**			
**Target population and subgroups**	4	Describe characteristics of the base case population and subgroups analysed, including why they were chosen.	44-46, Table 1
**Setting and location**	5	State relevant aspects of the system(s) in which the decision(s) need(s) to be made.	46-48
**Study perspective**	6	Describe the perspective of the study and relate this to the costs being evaluated.	49,50
**Comparators**	7	Describe the interventions or strategies being compared and state why they were chosen.	97-122
**Time horizon**	8	State the time horizon(s) over which costs and consequences are being evaluated and say why appropriate.	46,47
**Discount rate**	9	Report the choice of discount rate(s) used for costs and outcomes and say why appropriate.	94,95
**Choice of health outcomes**	10	Describe what outcomes were used as the measure(s) of benefit in the evaluation and their relevance for the type of analysis performed.	46-49, 136-141
**Measurement of effectiveness**	11a	*Single study-based estimates:* Describe fully the design features of the single effectiveness study and why the single study was a sufficient source of clinical effectiveness data.	NA
	11b	*Synthesis-based estimates:* Describe fully the methods used for identification of included studies and synthesis of clinical effectiveness data.	60-71, Table 1, Webappendix Table S7
**Measurement and valuation of preference based outcomes**	12	If applicable, describe the population and methods used to elicit preferences for outcomes.	NA
**Estimating resources and costs**	13a	*Single study-based economic evaluation:* Describe approaches used to estimate resource use associated with the alternative interventions. Describe primary or secondary research methods for valuing each resource item in terms of its unit cost. Describe any adjustments made to approximate to opportunity costs.	NA
	13b	*Model-based economic evaluation:* Describe approaches and data sources used to estimate resource use associated with model health states. Describe primary or secondary research methods for valuing each resource item in terms of its unit cost. Describe any adjustments made to approximate to opportunity costs.	73-89, Table 1, Webappendix Table S8 - S10
**Currency, price date, and conversion**	14	Report the dates of the estimated resource quantities and unit costs. Describe methods for adjusting estimated unit costs to the year of reported costs if necessary. Describe methods for converting costs into a common currency base and the exchange rate.	88,89
**Choice of model**	15	Describe and give reasons for the specific type of decision-analytical model used. Providing a figure to show model structure is strongly recommended.	43-58, Figure 1, Webappendix 1-24, Webappendix Table S1
**Assumptions**	16	Describe all structural or other assumptions underpinning the decision-analytical model.	52-71, Figure 1
**Analytical methods**	17	Describe all analytical methods supporting the evaluation. This could include methods for dealing with skewed, missing, or censored data; extrapolation methods; methods for pooling data; approaches to validate or make adjustments (such as half cycle	43-71, 97-134

		corrections) to a model; and methods for handling population heterogeneity and uncertainty.	
**Results**			
**Study parameters**	18	Report the values, ranges, references, and, if used, probability distributions for all parameters. Report reasons or sources for distributions used to represent uncertainty where appropriate. Providing a table to show the input values is strongly recommended.	43-134, Table 1, Webappendix Table S2 - S6
**Incremental costs and outcomes**	19	For each intervention, report mean values for the main categories of estimated costs and outcomes of interest, as well as mean differences between the comparator groups. If applicable, report incremental cost-effectiveness ratios.	151-161, Table 2, Webappendix Table S11
**Characterising uncertainty**	20a	*Single study-based economic evaluation:* Describe the effects of sampling uncertainty for the estimated incremental cost and incremental effectiveness parameters, together with the impact of methodological assumptions (such as discount rate, study perspective).	NA
	20b	*Model-based economic evaluation:* Describe the effects on the results of uncertainty for all input parameters, and uncertainty related to the structure of the model and assumptions.	182-201, Figure 3, Webappendix Table S14-S16
**Characterising heterogeneity**	21	If applicable, report differences in costs, outcomes, or cost-effectiveness that can be explained by variations between subgroups of patients with different baseline characteristics or other observed variability in effects that are not reducible by more information.	151-174, Table 2, Figure 2, Webappendix Table S12-S13
**Discussion**			
**Study findings, limitations, generalisability, and current knowledge**	22	Summarise key study findings and describe how they support the conclusions reached. Discuss limitations and the generalisability of the findings and how the findings fit with current knowledge.	203-304
**Other**			
**Source of funding**	23	Describe how the study was funded and the role of the funder in the identification, design, conduct, and reporting of the analysis. Describe other non-monetary sources of support.	321
**Conflicts of interest**	24	Describe any potential for conflict of interest of study contributors in accordance with journal policy. In the absence of a journal policy, we recommend authors comply with International Committee of Medical Journal Editors recommendations.	323 - 331

References

[1] Kowal P, Chatterji S, Naidoo N, Biritwum R, Fan W, Lopez Ridaura R, et al. Data Resource Profile: The World Health Organization Study on global AGEing and adult health (SAGE). Int J Epidemiol 2012;41:1639–49. https://doi.org/10.1093/ije/dys210.

[2] WHO. Global Health Observatory data repository. Geneva: World Health Organization; 2018.

[3] Global Burden of Disease Collaborative Network. Global Burden of Disease Study 2017 (GBD 2017) Results. Seattle, United States: Institute for Health Metrics and Evaluation (IHME); 2018.

[4] Ueda P, Woodward M, Lu Y, Hajifathalian K, Al-Wotayan R, Aguilar-Salinas CA, et al. Laboratory-based and office-based risk scores and charts to predict 10-year risk of cardiovascular disease in 182 countries: a pooled analysis of prospective cohorts and health surveys. Lancet Diabetes Endocrinol 2017;5:196–213. https://doi.org/10.1016/S2213-8587(17)30015-3.

[5] Sonnenberg FA, Beck JR. Markov Models in Medical Decision Making: A Practical Guide. Med Decis Making 1993;13:322–38. https://doi.org/10.1177/0272989X9301300409.

[6] Bellows BK, Ruiz-Negrón N, Bibbins-Domingo K, King JB, Pletcher MJ, Moran AE, et al. Clinic-Based Strategies to Reach United States Million Hearts 2022 Blood Pressure Control Goals: A Simulation Study. Circ Cardiovasc Qual Outcomes 2019;12:e005624. https://doi.org/10.1161/CIRCOUTCOMES.118.005624.

[7] Huffman MD, Rao KD, Pichon-Riviere A, Zhao D, Harikrishnan S, Ramaiya K, et al. A Cross-Sectional Study of the Microeconomic Impact of Cardiovascular Disease Hospitalization in Four Low- and Middle-Income Countries. PLoS ONE 2011;6:e20821. https://doi.org/10.1371/journal.pone.0020821.

[8] Petty GW, Brown RD, Whisnant JP, Sicks JD, O'Fallon WM, Wiebers DO. Survival and recurrence after first cerebral infarction: A population-based study in Rochester, Minnesota, 1975 through 1989. Neurology 1998;50:208–16. https://doi.org/10.1212/WNL.50.1.208.

[9] Lin JK, Moran AE, Bibbins-Domingo K, Falase B, Pedroza Tobias A, Mandke CN, et al. Cost-effectiveness of a fixed-dose combination pill for secondary prevention of cardiovascular disease in China, India, Mexico, Nigeria, and South Africa: a modelling study. Lancet Glob Health 2019;7:e1346–58. https://doi.org/10.1016/S2214-109X(19)30339-0.

[10] Smolina K, Wright FL, Rayner M, Goldacre MJ. Long-Term Survival and Recurrence After Acute Myocardial Infarction in England, 2004 to 2010. Circ Cardiovasc Qual Outcomes 2012;5:532–40. https://doi.org/10.1161/CIRCOUTCOMES.111.964700.

[11] Law MR, Morris JK, Wald NJ. Use of blood pressure lowering drugs in the prevention of cardiovascular disease: meta-analysis of 147 randomised trials in the context of expectations from prospective epidemiological studies. BMJ 2009;338:b1665. https://doi.org/10.1136/bmj.b1665.

[12] India Hypertension Management Intiative. Punjab Hypertension Protocol n.d.

[13] Chow CK, Teo KK, Rangarajan S, Islam S, Gupta R, Avezum A, et al. Prevalence, Awareness, Treatment, and Control of Hypertension in Rural and Urban Communities in High-, Middle-, and Low-Income Countries. JAMA 2013;310:959–68. https://doi.org/10.1001/jama.2013.184182.

[14] Rau N, Nayak SK. Practical Guidelines for Hypertension Management. Med. Update 2013, Mumbai, India: The Association of Physicians of India; 2013, p. 69–73.

[15] Jaffe MG, Frieden TR, Campbell NRC, Matsushita K, Appel LJ, Lackland DT, et al. Recommended treatment protocols to improve management of hypertension globally: A statement by Resolve to Save Lives and the World Hypertension League (WHL). J Clin Hypertens 2018;20:829–36. https://doi.org/10.1111/jch.13280.

[16] Van Wijk BL, Klungel OH, Heerdink ER, de Boer A. Rate and determinants of 10-year persistence with antihypertensive drugs: J Hypertens 2005;23:2101–7. https://doi.org/10.1097/01.hjh.0000187261.40190.2e.

[17] Dennis T, Meera NK, Binny K, Sekhar MS, Kishore G, Sasidharan S. Medication adherence and associated barriers in hypertension management in India. CVD Prev Control 2011;6:9–13. https://doi.org/10.1016/j.cvdpc.2010.11.001.

[18] Cherry SB, Benner JS, Hussein MA, Tang SSK, Nichol MB. The Clinical and Economic Burden of Nonadherence with Antihypertensive and Lipid-Lowering Therapy in Hypertensive Patients. Value Health 2009;12:489–97. https://doi.org/10.1111/j.1524-4733.2008.00447.x.

[19] TSMSIDC. Medicines-Rate Contract. Telangana State Med Serv Infrastruct Dev Corp n.d. http://tsmsidc.telangana.gov.in/content.php?U=20%20&&%20T=Medicines-Rate%20Contract (accessed January 7, 2020).

[20] WHO, Health Action International. Measuring price components. Meas. Med. Prices Availab. Affordabil. Price Compon. 2nd Ed., World Health Organization; n.d., p. 129–70.

[21] 1mg. About 1mg: India's leading digital consumer healthcare platform 2020. https://www.1mg.com/aboutUs (accessed May 15, 2020).

[22] Prinja S, Chauhan AS, Bahuguna P, Selvaraj S, Muraleedharan VR, Sundararaman T. Cost of Delivering Secondary Healthcare Through the Public Sector in India. PharmacoEconomics 2020;4:249–61. https://doi.org/10.1007/s41669-019-00176-9.
[23] Central Government Health Scheme, GoI. Rate List for Hyderabad - CGHS Hyderabad w.e.f 17/11/2014. Hyderabad, India: Government of India; 2014.
[24] WHO. Country-specific inpatient and outpatient estimates in 2010 currency. Geneva: World Health Organization; 2010.
[25] Xavier D, Pais P, Devereaux P, Xie C, Prabhakaran D, Reddy KS, et al. Treatment and outcomes of acute coronary syndromes in India (CREATE): a prospective analysis of registry data. The Lancet 2008;371:1435–42. https://doi.org/10.1016/S0140-6736(08)60623-6.
[26] MSH. International Medical Products Price Guide. Arlington,Virginia: Management Sciences for Health; 2016.
[27] Husereau D, Drummond M, Petrou S, Carswell C, Moher D, Greenberg D, et al. Consolidated Health Economic Evaluation Reporting Standards (CHEERS) statement. BMJ 2013;346:f1049. https://doi.org/10.1136/bmj.f1049.
